# Nobiletin inhibits breast cancer via p38 mitogen-activated protein kinase, nuclear transcription factor-κB, and nuclear factor erythroid 2-related factor 2 pathways in MCF-7 cells

**DOI:** 10.29219/fnr.v62.1323

**Published:** 2018-11-21

**Authors:** Jianli Liu, Shuai Wang, Siqi Tian, Yin He, Hong Lou, Zhijun Yang, Yuchi Kong, Xiangyu Cao

**Affiliations:** School of Life Science, Liaoning University, Shenyang, China

**Keywords:** nobiletin, breast cancer MCF-7 cells, anticancer, apoptosis, cell signaling pathway

## Abstract

**Introduction:**

Breast cancer is one of the most commonly diagnosed cancers in women, with a high mortality rate.

**Objective:**

In the present study, we evaluated the anticancer effect of nobiletin, a flavone glycoside, on the breast cancer cell line MCF-7.

**Result:**

Cell viability and proliferation decreased and cell morphology changed from diamond to round after being treated with nobiletin. Nobiletin induced apoptosis of breast cancer MCF-7 cells via regulating the protein expression of Bax, Bcl-2, cleaved caspase-3, and p53. The expression of Bcl-2 decreased, while the expression of Bax and p53 increased in MCF-7 cells treated with nobiletin. Meanwhile, nobiletin inhibited cell migration by downregulating the protein expression of matrix metalloproteinase-2 (MMP-2) and matrix metalloproteinase-9 (MMP-9). Moreover, phosphorylation of p38 was increased, and the translocation of p65 and nuclear factor erythroid 2-related factor 2 (Nrf2) to the nucleus was decreased, which suggested that the anticancer effects of nobiletin might at least partially rely on mediating the p38 mitogen-activated protein kinase, nuclear transcription factor-κB, and Nrf2 pathways in MCF-7 breast cancer cells.

**Conclusion and recommendation:**

Our data showed that nobiletin was a potential antitumor drug, and it provided some experimental basis for the clinical application of tumor therapy.

## Highlights

- Nobiletin induced apoptosis of MCF-7 human breast cancer cells.
- Nobiletin suppressed colony formation and migration of MCF-7 human breast cancer cells.
- Potential molecular mechanism of nobiletin against MCF-7 cells may rely on mediating p38 MAPK, NF-κB, and Nrf2 pathways.

Breast cancer is one of the most commonly diagnosed cancers in the world, and it has a high frequency and high mortality rate in women, especially African-American women (1). A Chinese Health Bureau statistics report showed that the current incidence of breast cancer had become the primary cancer in Chinese women. Researchers have confirmed some of the risk factors for breast cancer, including penetrant gene mutations (2), genetic polymorphisms (3), and breast age (4). Recent researches showed that some bioactive compounds extracted from plants, such as valproic acid (5), berberine (6), and galangin (7), could induce apoptosis of breast cancer cell lines. Obviously, it is important to find new products to inhibit tumorigenesis.

Nobiletin is extracted from *Citrus nobilis* Lour., *C. aurantium* L, and *C. reticulata* Blanco and has been applied for antiagglutination, antithrombosis, and anti-inflammatory uses. Recently, it was reported that nobiletin played an antitumor role. Nobiletin inhibits tumorigenesis and induces apoptosis of human cancer cells, including human osteosarcoma cells (8), human fibrosarcoma HT-1080 cells (9), and colorectal cancer cells (10). Nobiletin decreased the levels of phospho-ERK2 and phospho-AKT to attenuate metastasis in human cancer HepG2 cells (11). Thus, nobiletin is regarded as a promising chemotherapeutic drug for cancer treatment. It also has been reported that dietary flavonoid nobiletin could induce its own metabolism and in turn enhance its cytostatic effect in MCF7 breast cancer cells, by cytochrome P450-1A1 (CYP1A1) and cytochrome P450-1B1 (CYP1B1) upregulation (12).

Cell apoptosis plays an important role in the germination and growth of tumors (13). Recent studies have shown that p38 mitogen-activated protein kinase (MAPK) is vital to the apoptosis of tumor cells (14). It is obvious that the mechanism of tumor cell apoptosis is mediated by the p38 MAPK signal transduction pathway under the action of different stimuli, including induction of apoptosis through caspase-dependent apoptotic pathways (15), induction of apoptosis by phosphorylation of p53, as well as induction of apoptosis by members of the Bcl-2 protein family (16). It has been reported that ginkgetin inhibited several human breast cancer cell lines by regulating the MAPK pathway (13). In most tumor cell types, nuclear transcription factor-κB (NF-κB) is in a state of continuous activation; by contrast it is inactive and retained in the cytoplasm in most normal cells and is released and translocated to the nucleus when activated (17). Inhibition of the NF-κB pathway in tumor cells can block the cell cycle and induce cell apoptosis (18). Thus, the NF-κB pathway plays an important role in tumor proliferation. According to Z. Yuan (19), activation of NF-κB has been found in breast cancer repeatedly and leads to overexpression of downstream signaling targets, for example anti-apoptotic genes, to strengthen growth and chemoresistance (20). Nuclear actor erythroid-2-related factor 2 (Nrf2) is an important defense signaling pathway in the development of tumors, participating in anti-inflammatory activities, apoptosis, and tumorigenesis (21). In tumor cells, it has been reported that Nrf2 activity is inhibited by blocking Nrf2 protein transfer from the cytoplasm into the nucleus, which makes cancer resistant to chemotherapeutic drugs and inhibits the occurrence of apoptosis (22).

The antitumor effect of nobiletin has been studied in human cancer cell lines, but the potential anticancer activity of nobiletin against breast cancer cells is unknown, owing to a lack of research. An *in vitro* model of MCF-7 human breast cancer cells was developed in a previous study, which allowed us to evaluate its impact at the cellular level and determine the ability of this compound for apoptosis, cell proliferation, and migration. It furthermore enabled us to understand the role of the p38 MAPK, NF-κB, and Nrf2 signaling pathways on the antitumor activity of nobiletin. Thus, the antitumor effect of nobiletin and its probable mechanism in breast cancer were investigated in the present study.

## Materials and methods

### Chemicals and reagents

Nobiletin (purity >98%) was purchased from Chengdu Must Biotechnology Co., Ltd. (Chengdu, China). Nobiletin was dissolved in dimethyl sulfoxide (DMSO) and the final chroma of DMSO in the cell culture was kept below 0.05%. Phosphate buffered saline (PBS), protease inhibitor cocktail, and bicinchoninic acid (BCA) assay kit were purchased from Dingguo Changsheng Biotechnology (Beijing, China). Further, 3-(4,5-dimethylthiazol-2-yl)-2,5-diphenyltetrazolium bromide (MTT) and nuclear extraction kit were purchased from Sigma Aldrich (St. Louis, MO, USA). Sodium dodecyl sulfate (SDS) was purchased from Sinopharm Chemical Reagent Co., Ltd. (Shanghai, China). Annexin V-FITC Apoptosis Detection Kit and Hoechst 33258 were purchased from Nanjing KeyGen Biotech Co., Ltd. (Nanjing, China). The primary antibodies for MMP-2, MMP-9, p-p38, p38, Nrf2, NF-κB, Bax, Bcl-2, p53, caspase-3, anti-proliferating cell nuclear antigen (PCNA), β-actin and all secondary antibodies were purchased from Cell Signaling Technology (Boston, MA, USA). Streptomycin, penicillin, DMEM medium and fetal bovine serum (FBS) were purchased from Hyclone (Logan, UT, USA).

### Cell culture

MCF-7 human breast cancer cells were obtained from the Cell Bank of Type Culture Collection of the Chinese Academy of Sciences (Shanghai, China). MCF-7 cells were cultured in high-glucose DMEM supplemented with 10% FBS and antibiotics (penicillin 100 U/mL and streptomycin 100 μg/mL). Culture conditions were maintained in an incubator at 37°C containing 5% CO_2_.

### Cell morphological observation

MCF-7 cells were seeded in 12-well plates at a density of 3 × 10^5^ cells/well. After treatment with different concentrations of nobiletin (50, 100, and 200 μM) for 24 h and 48 h, the cell morphology of each group was observed by Olympus CX22LED microscope (10× magnification, Olympus Corporation, Tokyo, Japan).

### Cell growth inhibition assay

As described previously (23), MTT assay was used to measure cell viability. Briefly, cells (3 × 10^3^ cell/well) were seeded in 96-well plates and treated with nobiletin (12.5, 25, 50, 100, and 200 μM) in 200 mL cell medium for 24 h. Five replicates were prepared for each dose group. After nobiletin treatment, the medium was removed and fresh medium with MTT (0.5 mg/mL) was added, followed by incubation at 37°C for 4 h in a CO_2_ incubator. After brief centrifugation, supernatants were carefully removed and DMSO was added to dissolve the formazan crystals. After the formazan crystals were completely dissolved, the absorbance at 490 nm was determined with a microplate reader (Multiskan FC, Thermo Fisher Scientific, St. Herblain, France).

### Annexin V-fluorescein isothiocyanate/propidium iodide double staining test

Annexin V-fluorescein isothiocyanate (V-FITC) and propidium iodide (PI) double staining was applied to detect the apoptosis rate of the cells (24). In short, MCF-7 cells were seeded in 6-well plates at a concentration of 1 × 10^6^ cells/well, cultured for 24 h, and treated with nobiletin (50, 100, and 200 μM) on the following day. Then, MCF-7 cells were collected, washed twice using cold PBS buffer, and suspended with annexin V (5 μL) and PI (5 μL) solution in 500 μL binding buffer for 15 min at room temperature. The cell apoptosis rate was analyzed via flow cytometry (FACSCalibur, BD, New Jersey, Franklin Lake, USA.

### Hoechst 33258 staining

MCF-7 cells were seeded on coverslips placed in the flat bottom of 12-well plates and treated with nobiletin (50, 100, and 200 μM) in 2 mL cell medium for 24 h. Three replicates were prepared for each dose group. After being washed with PBS twice, the cells on the coverslips were fixed with 4% paraformaldehyde solution for 30 min, and fluorescent staining was conducted with 30 μM of Hoechst 33258 for 15 min at room temperature. Chromatin condensation of the cells was observed under a fluorescence microscope at 340 nm (20× magnification).

### Wound healing assay

In order to determine the inhibition of nobiletin on MCF-7 cell line migration (25), a wound healing assay was performed in 6-well plates. After the cells reached over 75% confluency, a sterile micropipette tip was used to scrape the cells. After scraping, the cells were washed with PBS twice. The cells were exposed to nobiletin (50, 100, and 200 μM) for 24 h. Meanwhile, the same fields of the wound migration were photographed (10× magnification) at 24 h and 48 h.

### Western blot analysis

MCF-7 cells were harvested and lysed, and total protein extracts and nuclear extracts were prepared with total protein and nuclear protein extraction kits. The protein concentration of the extracts was estimated using BCA protein assay according to the manufacturer’s instructions. Equal proteins were separated by SDS polyacrylamide gel electrophoresis and transferred to polyvinylidene fluoride membranes. After blocking with 5% nonfat milk for 1 h, the blots were incubated with primary antibodies overnight at 4°C. The following primary antibodies were used: anticaspase-3 antibody, anti-p53 antibody, anti-Bax antibody, anti-Bcl-2 antibody, anti-MMP-9 antibody, anti-MMP-2 antibody, anti-p38 antibody, anti-phospho-p38 antibody, anti-NF-κB antibody, anti-Nrf2 antibody, anti-PCNA antibody, and anti-β-actin antibody. The membranes were washed with TBST buffer and incubated with the secondary antibodies. Finally, the blotswere visualized using enhanced chemiluminescence detection reagents. The relative protein levels were normalized to β-actin or anti-proliferating cell nuclear antigen (PCNA) intensity.

### Statistical analysis

All data were presented as mean ± SD for at least three independently performed experiments. Gray intensity was analyzed using Image J software. Statistical analyses were carried out with Student’s tests and analysis of variance using the software SPSS version 22 (SPSS, Inc., Chicago, IL, USA). Differences with *p* < 0.05 and *p* < 0.01 were considered to be statistically significant.

## Results

### Effect of nobiletin on cell morphology in MCF-7 cells

**Fig. 1:**
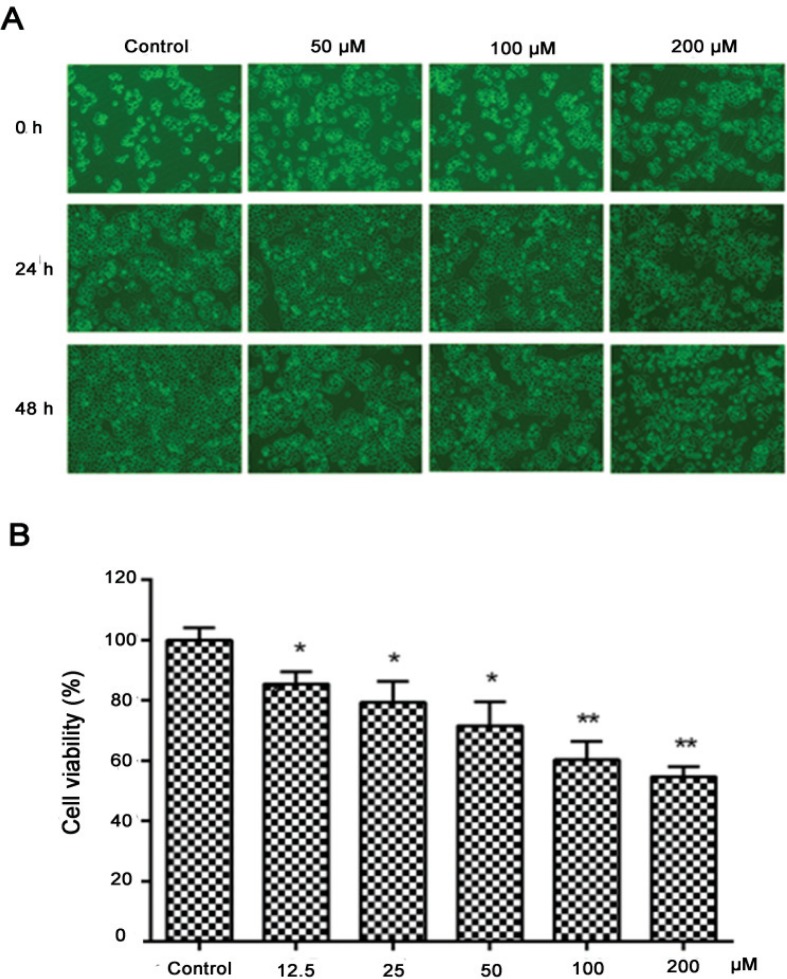
Effects of nobiletin on cell morphology and viability of MCF-7 cells. (A) MCF-7 cells were cultured with various concentrations (50, 100, and 200 μM) of nobiletin for 24 and 48 h and the images were obtained (10×). (B) Cell viability was determined by MTT assay. Compared with the control group, the survival rate of the cells in the dosage group after treatment was higher. Data are expressed as means ± SD from three independent experiments. *p < 0.05 compared with their respective control cells. **p < 0.01 compared with their respective control cells.

Through a microscope, the cells were in good condition and adhered firmly in the control group, while morphological changes were observed in the treated groups. After being treated with nobiletin (50, 100, and 200 μM) for 24 h or 48 h, MCF-7 cells were observed shrinking and their adhesion weakened, even floating in the culture medium in a concentration-dependent manner (Fig. 1A).

### Effect of nobiletin on cell viability in MCF-7 cells

An MTT assay was conducted to evaluate the effect of nobiletin on the cell viability of MCF-7 cells. Cells were treated with nobiletin at different concentrations for 24 h. As shown in Fig. 1B, nobiletin decreased the cell viability to 85.3 ± 4.5% (*p* < 0.05), 79.3 ± 7.0% (*p* < 0.05), 71.2 ± 5.0% (*p* < 0.05), 60.3 ± 6.0% (*p* < 0.01), and 54.4 ± 4.5% (*p* < 0.01) of the control at different concentrations (12.5, 25, 50, 100, and 200 μM). These results showed that nobiletin significantly (*p* < 0.05) decreased the viability of MCF-7 cells and appeared to inhibit cell growth in a dose-dependent manner.

### Effect of nobiletin on migration in MCF-7 cells

**Fig. 2:**
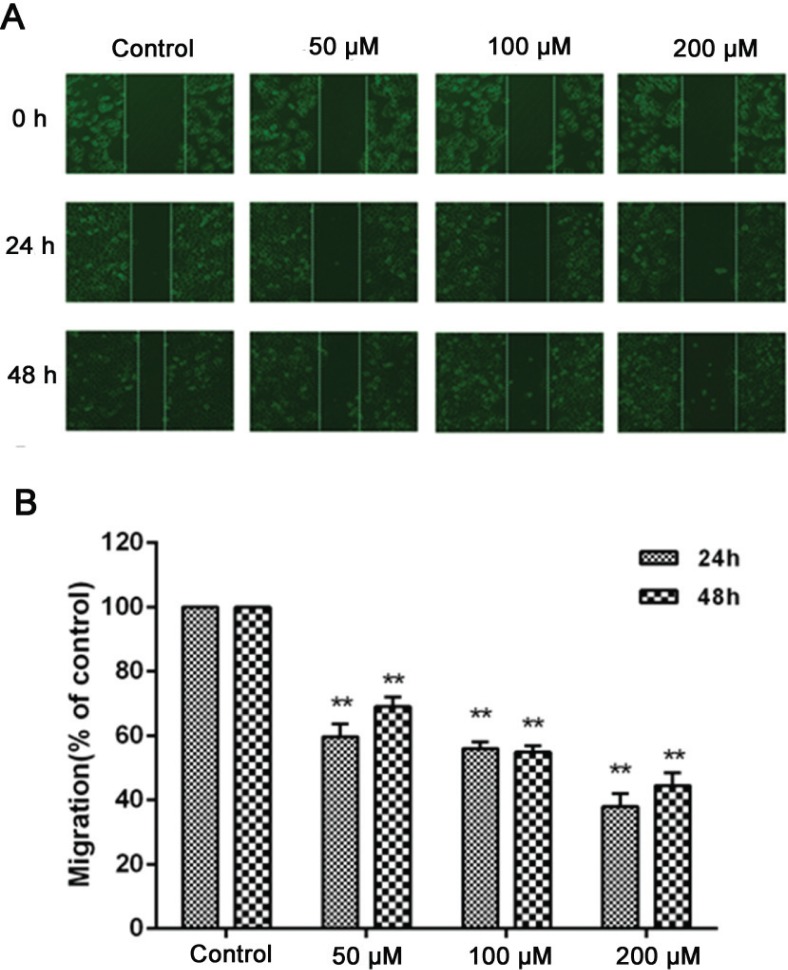
Effect of nobiletin on migration of MCF-7 cells. Cell migration was observed with a microscope (10×) at 24 and 48 h and the relative migration was calculated. (A) MCF-7 cells were seeded in 12-well plates, and the cell layer was scratched with a 10 μl sterile tip. (B) Cell migration was observed with a microscope (magnification, ×10) at 24 and 48 h. Data represented as mean ± SD of three independent experiments. **p < 0.01 compared with their respective controlcells.

Cancer cell migration is vital for tumor growth and prognosis. Therefore, we observed the effect of nobiletin on the migration ability of MCF-7 cells by wound healing assay. The results showed that compared to the control group, nobiletin inhibited the narrowing of the defect area in a dose-dependent manner. When the nobiletin concentration reached 200 μM, the inhibition rates were 38.2 ± 3.2% and 44.8 ± 2.5% at 24 h and 48 h, indicating that nobiletin has a significant inhibitory effect on migration ability (Fig. 2).

### Effect of nobiletin on apoptosis in MCF-7 cells

**Fig. 3:**
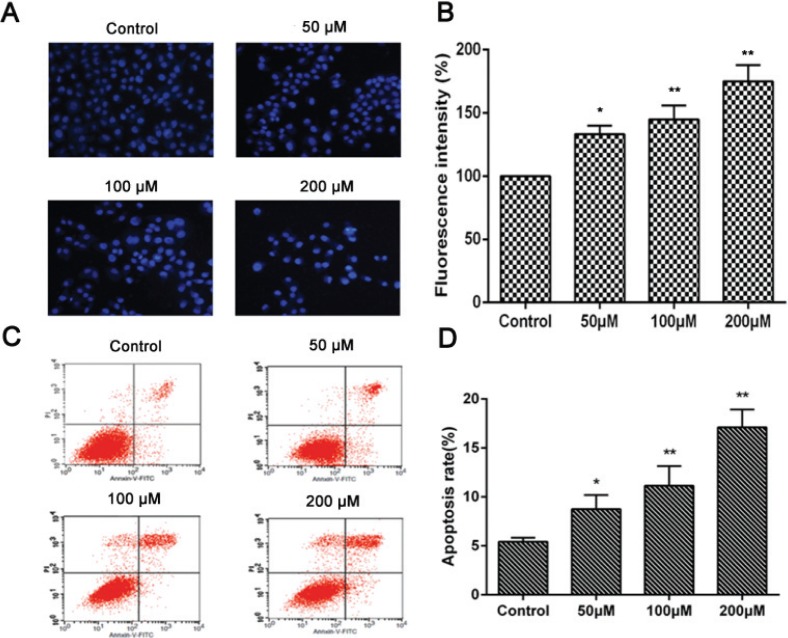
Induction of apoptosis in MCF-7 cells with Nobiletin.(A) Fluorescence images and (B) fluorescence intensity of MCF-7 cells subjected to Hoechst 33258 staining of nuclei following treatment with various concentrations of Nobiletin for 24 h (magnification, ×20) (C) Flow cytometry and (D) percentage of apoptotic cells examined by Annexin V-FITC and PI assay following treatment with various concentrations of Nobiletin for 24 h. Data represented as mean ± SD of three independent experiments. *p < 0.05 compared with control cells. **p < 0.01 compared with their respective control cells.

The main types of cell death include necrosis, autophagic cell death, and apoptosis (26). We performed a Hoechst 33258 staining assay to study which type of cell death was induced by nobiletin in MCF-7 cells. As shown in Fig. 3A and B, cells treated with nobiletin partially displayed apoptotic morphological changes in their nuclei, including apoptotic bodies and nuclear condensation. Furthermore, the double labeling assay obtained a quantitative analysis of different periods of cell apoptosis, including early apoptotic cells and terminal apoptotic cells by flow cytometry assay (Fig. 3C and D). The results showed that nobiletin (50, 100, and 200 μM) induced cell apoptosis at the rates of 8.62 ± 3.5% (*p* < 0.05), 11.2 ± 2.0% (*p* < 0.01), and 17.1 ± 3.7% (*p* < 0.01), respectively, which were significantly higher than the percentage of apoptosis in the untreated cells (5.4 ± 0.97%). In addition, the percentages of apoptotic cells clearly increased with increasing concentrations of nobiletin, showing a clear dose–effect relationship. The highest apoptotic rate of 17.1% was observed when nobiletin was administered at a concentration of 200 μM (Fig. 3D). These results indicate that the induction of apoptosis was a major effect of nobiletin on MCF-7 cells.

### Effect of nobiletin on expression of MMP-2 and MMP-9

**Fig. 4:**
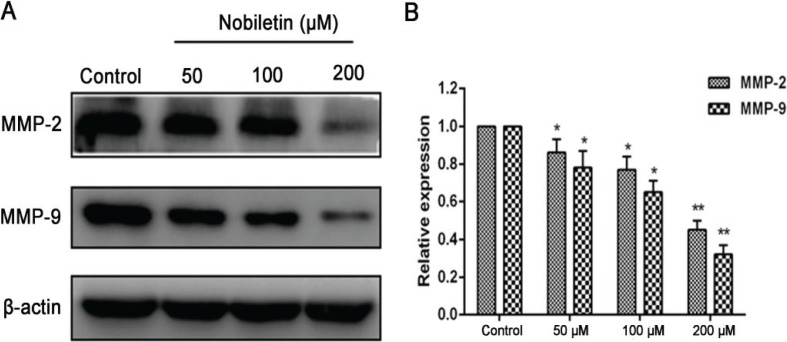
The effect of nobiletin on protein expression of MMP-2 and MMP-9 in MCF-7 treated with nobiletin (50, 100, and 200 μM). (A) Western blots and (B) quantified protein expression levels of MMP-2, MMP-9 and β-actin. Data represented as mean ± SD of three independent experiments. *p < 0.05 compared to their respective control cells. **p < 0.01 compared to their respective control cells.

MMPs, which are involved in the degradation of the basement membrane, are essential to the migratory process (27). To examine the effect of nobiletin on MMP levels, MCF-7 cells were treated with 50, 100, and 200 μM nobiletin for 24 h. Last, the protein expression of MMP-2 and MMP-9 in each group was measured by Western blot assay. The results showed that expression levels of MMP-2 and MMP-9 were significantly lower (*p* < 0.01) than those in the control group of the MCF-7 cell line (Fig. 4).

### Effect of nobiletin on expression of apoptosis-related proteins

**Fig. 5:**
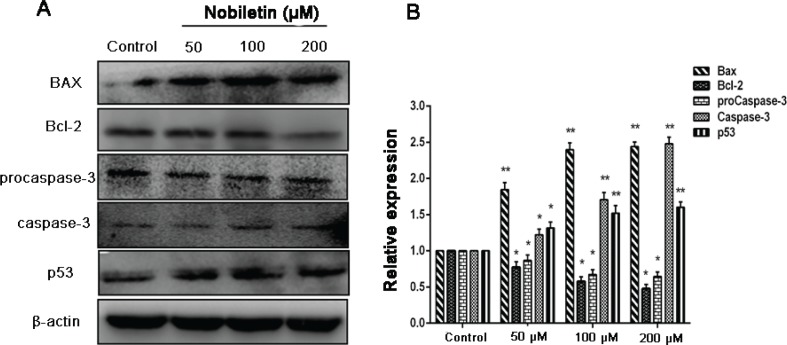
Effect of nobiletin on protein expression of Bax, Bcl-2, p53, and caspase-3 in MCF-7 cells. (A) Western blots and (B) quantified protein expression levels of Bax, Bcl-2, procaspase-3, caspase-3, p53 and β-actin Data represented asmean± SD of three independent experiments. *p < 0.05 compared to their respective control cells. **p < 0.01 compared with their respective control cells.

Bcl-2 family members play an important role in apoptosis (28), especially Bax and Bcl-2 proteins, which form a heterodimeric complex, thereby neutralizing its apoptotic effects. At the same time, the protein p53 is also related to apoptosis (29, 30). We observed that treatment of cells with nobiletin resulted in a significant (*p* < 0.05) decrease in Bcl-2 expression with a significant (*p* < 0.05) increase in the protein level of Bax and p53 (Fig. 5). In most cancer cells, caspases require site-specific cleavage of the protein to become active and participate in the process of apoptosis (14). To evaluate whether caspases are involved in apoptosis induction by nobiletin, we detected the expression levels of procaspase-3 and caspase-3 proteins. The results showed that the levels of procaspase-3 expression was significantly (*p* < 0.05) decreased when treated with 50, 100, and 200 μM, while the levels of caspase-3 expression in MCF-7 cells increased significantly (*p* < 0.05) compared with the control group.

### Effect of nobiletin on the NF-κB pathway

**Fig. 6:**
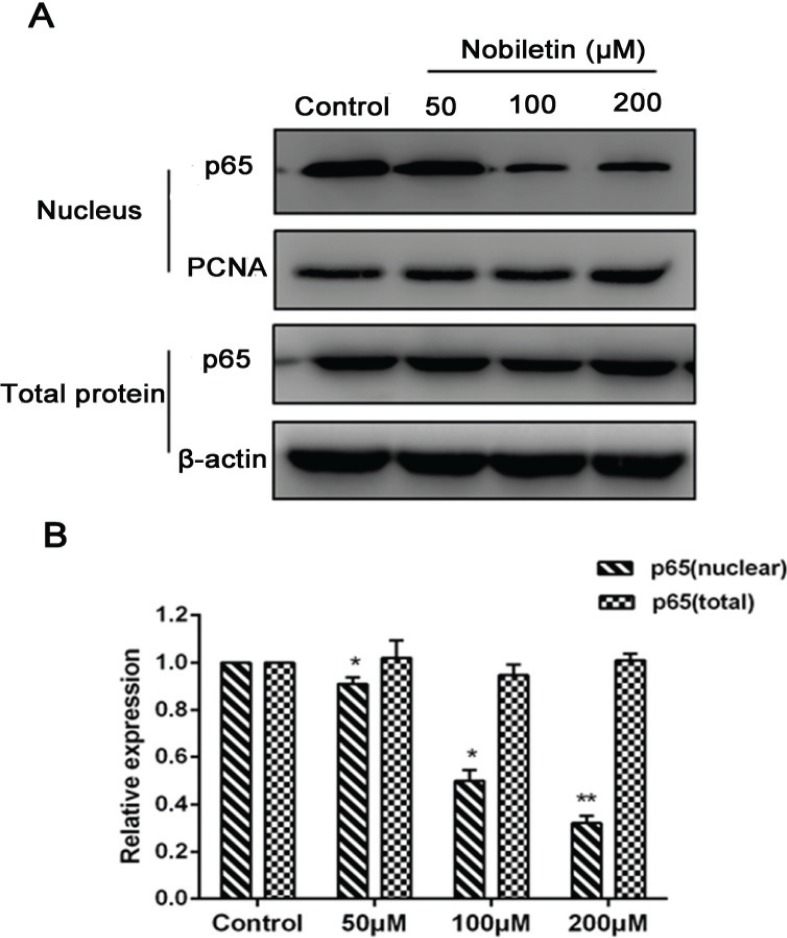
Effects of nobiletin on protein expression of p65 (nuclear and cytoplasmic) in MCF-7 cells. (A) Western blots, and quantified protein levels of (B) total-p65 and nuclear-p65. Data represented as mean ± SD of three independent experiments. *p < 0.05 compared to their respective control cells. **p < 0.01 compared with their respective control cells.

The protein expressions of p65 in each group were measured to explore the contribution of the NF-κB pathway to the antitumor effect of nobiletin on MCF-7 cells. As shown in Fig. 6, 50, 100, and 200 μM nobiletin significantly (*p* < 0.05) downregulated the translocation of p65 in the nuclei of MCF-7 cells; however, nobiletin at these concentrations could not significantly (*p* > 0.05) affect the protein expression of p65 in cytoplasmic extract.

### Effect of nobiletin on the p38 MAPK pathway

**Fig. 7:**
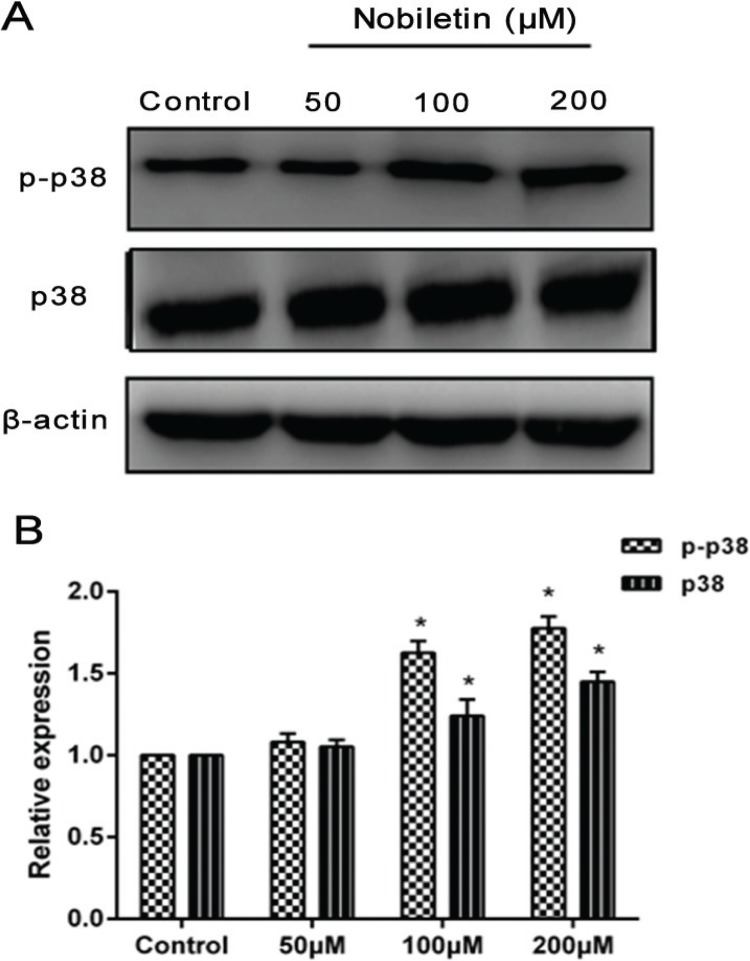
Effects of nobiletin on protein expression of p38 and p-p38 in MCF-7 cells. (A) Western blot analyses and (B) quantified levels of p38 and p-p38 in MCF-7 cells treated with different concentrations of Nobiletin for 24 h. Data represented as mean ± SD of three independent experiments. *p < 0.05 compared with control cells. **p < 0.01 compared with their respective control cells.

P38 MAPK signaling pathways, which are found in all eukaryotes, have been demonstrated to play a central role in diverse biological processes, such as cell proliferation and apoptosis (31). To go a step further and evaluate the mechanism by which nobiletin induced damage in the MCF-7 cells, we measured the protein expression of p38 and phosphorylation of p38 in each group. After treatment with nobiletin (100 and 200 μM), the levels of p-p38 were significantly higher (*p* < 0.05) compared with those in the control group, but 50 μM nobiletin had no obvious (*p* > 0.05) effect on the phosphorylation of p38. Moreover, 100 and 200 μM nobiletin had an obvious (*p* < 0.05) effect on the expression of p38 compared with that in the control groups (Fig. 7).

### Effect of nobiletin on Nrf2 pathway

**Fig. 8:**
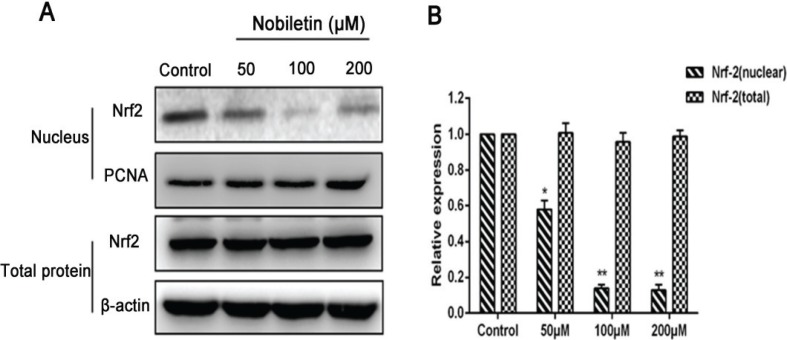
Effects of nobiletin on protein expression of Nrf2 (nuclear and cytoplasmic) in MCF-7 cells line. (A) Western blots and (B) quantified protein expression levels of total-Nrf2 and nuclear-Nrf2.Data represented as mean ± SD of three independent experiments. *p < 0.05 compared with control cells. **p < 0.01 compared with their respective control cells.

Nrf2 downregulation is a potential therapeutic approach in cancer treatment (32). MCF-7cells were damaged after being incubated with nobiletin. After treatment with 100 and 200 μM nobiletin, we measured the protein expression of Nrf2 in each group. The levels of Nrf2 were significantly (*p <* 0.05) decreased in the nuclei compared to those in the control groups of MCF-7 cells, but 50 μM nobiletin had no obvious (*p* > 0.05) effect. However, the various concentrations of nobiletin did not significantly (*p* > 0.05) affect the protein expression of Nrf2 in cytoplasmic extract (Fig. 8).

## Discussion

Breast cancer is one of the most commonly diagnosed cancers, with a high mortality rate in women (33). Thus, it is vital to find anti–breast cancer drugs. In this study, we explored the effect of nobiletin on MCF-7 breast cancer cells by measuring markers of proliferation, apoptosis, and migration with positive results and carried out preliminary research about its mechanisms.

To research whether nobiletin could induce damage in MCF-7 cells, we observed cell morphology and measured cell viability. The results showed that nobiletin weakened cell adhesion and significantly decreased the viability of MCF-7 cells; moreover, cell morphology changed from diamond to round after being treated with nobiletin, suggesting that nobiletin caused cell damage and inhibited the proliferation of MCF-7 cells.

Apoptosis is an essential mechanism for cell death following many types of chemotherapy. In the present study, cell apoptosis was determined by Hoechst 33258 staining and annexin V/PI double staining. The results showed that nobiletin significantly increased the apoptotic rates of MCF-7 cells, suggesting that nobiletin induced apoptosis in breast cancer cells. The mitochondrial (or intrinsic) apoptosis pathway is mainly regulated by the interplay between members of the Bcl-2 protein family (28). When the expression of Bax is upregulated, cytochrome C is released from the mitochondria to the cytoplasm because of mitochondrial outer membrane permeability, followed by apoptosis complex and caspase activation (34). We observed that nobiletin significantly downregulated Bcl-2 protein, upregulated levels of Bax protein, and upregulated levels of caspase-3 protein in MCF-7 cells, suggesting the involvement of an intrinsic apoptotic pathway by which nobiletin induces apoptosis in MCF-7 cells. The protein p53 was identified as the common mutated tumor suppressor in human tumorigenesis (29). Moreover, the protein p53 is also related to cell cycle progression, DNA repair, and apoptosis (30). Therefore, some studies have focused on p53 mediating the effect of drug intervention in initiating apoptotic death. Importantly, a histopathological analysis of the p53 Cre-loxP mouse revealed no signs of p53-mediated toxicity in normal organs after p53 activation, suggesting that p53 activation could be a method of tumor clearance with limited toxic side effects to normal tissues (35). In this study, we found that the expression of p53 was increased in MCF-7 cells by treatment with nobiletin, which further suggested that p53 may play an important role in the apoptosis of MCF-7 cells, and nobiletin may induce cell apoptosis through intrinsic apoptotic pathways.

The high lethality of the major cancers is owing to the diffusion of metastatic cancer cells and the growth of tumors at distant sites (36). To further evaluate the nobiletin for anticancer effect on breast cancer cells, we studied the effect of nobiletin on MCF-7 cell lines towards proliferation and migration, which are related to development of therioma. We found that proliferation and migration of breast cancer cells were significantly inhibited by treatment with nobiletin. The tumor cell model of breast cancer has shown that increased MMP expression is associated with increased tumor cell growth and metastasis potential (37). Among the MMPs, MMP-9 and MMP-2 are abundantly expressed in various malignant tumors and are considered to play critical roles in tumor metastasis (38). It has been reported that plumbagin may inhibit the migration of glioma cells through reduction of the protein levels of MMP-2 and MMP-9 (39). Moreover, MMPs are continually overexpressed in almost all human cancer cells and are regarded as potential targets of cancer therapy (40). Thus, to study the mechanisms of the inhibitive effect of nobiletin on migration of breast cancer cells, we measured the expression of MMP-2 and MMP-9. Our results showed that nobiletin significantly inhibited the levels of MMP-2 and MMP-9 in MCF-7 cells, indicating that nobiletin may inhibit migration partially by deregulating the expression of MMPs.

Tumorigenesis is involved with multiple factors and multimechanisms; a series of changes in signaling transduction on molecules can be observed in this process. The MAPK is related to occurrence of damage and development of resistance to drugs (41); MAPK is a complicated network system that plays an important role in cell proliferation, apoptosis, and metastasis (42). In particular, p38 MAPK is a vital signaling pathway that is responsible for the rate of cell proliferation, transfer, and apoptosis (43). The protein p38 can be activated when extracellular stimuli occur, specifically phosphorylation, and p-p38 is shuttled into the nucleus to activate transcription factors and promote apoptosis. Studies have demonstrated that pretreatment with the p38 MAPK inhibitor SB203580 obviously reduced the expression of p38 MAPK protein, as well as blocking tetrahydrocurcumin-mediated Bax upregulation, Bcl-2 downregulation, and caspase-3 activation (31). According to Jun Cao, blockage of p38 MAPK decreased antitumor activity of ginkgetin and suggested that p38 MAPK might be involved in ginkgetin-induced inhibitory effects on breast cancer cell growth (13). Our research showed that p38 MAPK was activated by nobiletin in MCF-7 breast cancer cells and suggested that p38 MAPK might be involved in nobiletin-induced inhibitory effects of cell migration and promote apoptosis in breast cancer cells. Moreover, MAPKs have been reported to regulate MMP-9 expression (44). MAPKs are associated with other signaling pathways; some studies have shown that NF-κB (p65) is a downstream target of p38 MAPK. NF-κB (p65) has been regarded as an important inducible transcription factor that plays an important role in cell growth, proliferation, apoptosis, and carcinogenesis. It has been reported that celecoxib-induced apoptosis against different breast cancer cell lines by downregulating the NF-κB pathway (45).

Moreover, when the NF-κB pathway was inhibited, reduction of migration and invasion were observed (17), as well as NF-κB transfer from the cytoplasm into the nucleus, which is a major step in regulating MMP transcription. In this study, we found that nobiletin significantly downregulated the level of NF-κB in the nuclei of breast cancer cells. Thus, we speculated that nobiletin might induce apoptosis and inhibit cell migration partly by precluding the translocation of NF-κB from the cytoplasm into the nucleus.

Nrf2 downregulation is deemed a potential therapeutic approach in cancer treatment. It has been reported that inhibition of Nrf2 by RNA interference knockdown can resensitize some cancer cells to anticancer drugs (46). According to I. Bobilev, vitamins downregulate Nrf2 translocation into the nucleus causing the death of cancer cells in leukemia (47). Cryptotanshinone inhibits cell proliferation and increases apoptosis of cancer cells, including human breast cancer (48) and colorectal cancer (49). Moreover, some flavonoids, including luteolin and chrysin, suppress Nrf2 activity via promoting Nrf2 protein degradation and sensitize cancer cells to chemotherapeutic drugs (22, 50). Our data showed that Nrf2 was inhibited from transferring into the nucleus under the effect of nobiletin on breast cancer MCF-7 cells.

## Conclusions

**Fig. 9:**
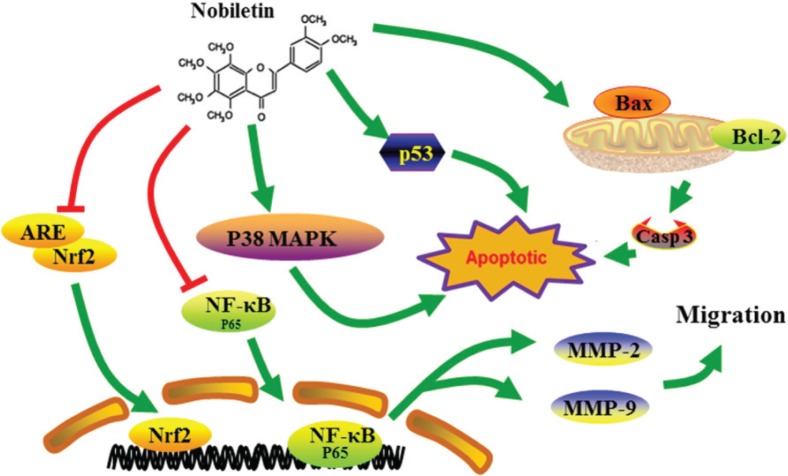
Nobiletin-induced apoptosis and suppressed migration and proliferation of human breast cancer cells via regulating p38 MAPK, NF-κB pathway, and Nrf2 pathway.

In summary, our results indicate that nobiletin inhibits the migration and proliferation of human breast cancer cells. Furthermore, nobiletin promoted apoptosis in breast cancer cells by regulating Bax/Bcl-2, caspase-3, and p53 expression. This might be associated with the participation of the NF-κB, p38 MAPK, and Nrf2 pathways (Fig. 9). Our study suggests that nobiletin is a promising anticancer drug candidate for breast cancer therapy.
