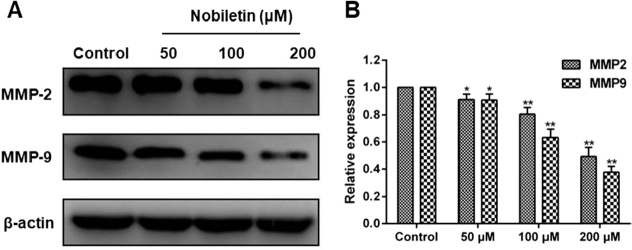# Nobiletin inhibits breast cancer via p38 mitogen-activated protein kinase, nuclear transcription factor-κB, and nuclear factor erythroid 2-related factor 2 pathways in MCF-7 cells

**DOI:** 10.29219/fnr.v69.12680

**Published:** 2025-06-30

**Authors:** Jianli Liu, Shuai Wang, Siqi Tian, Yin He, Hong Lou, Zhijun Yang, Yuchi Kong, Xiangyu Cao

**Affiliations:** School of Life Science, Liaoning University, Shenyang, China

In Figs. 3C and 4A of the published version, the Flow cytometry image of 50 μM Nobiletin in Fig. 3C and western blot band of MMP2 and MMP9 in Fig. 4A were presented incorrectly. The corrected Figures are provided as below. This corrigendum does not affect the accuracy of result and conclusion of this work. The authors regret for any inconvenience caused.

*Fig. 3:*

**Figure:**
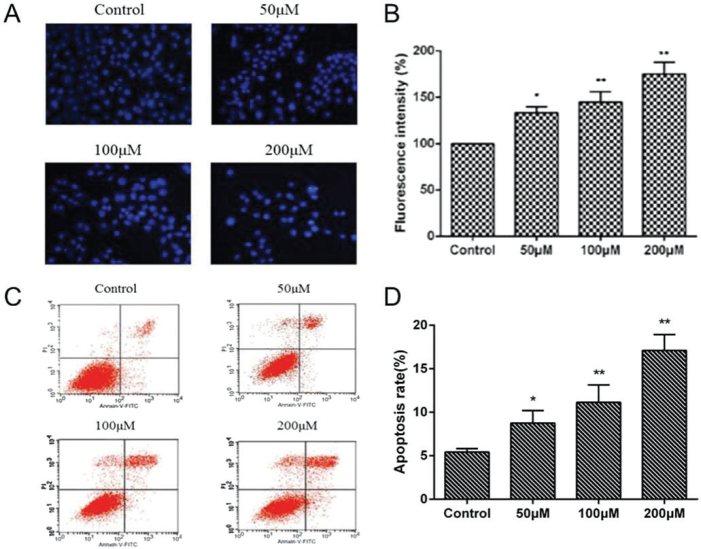


*Fig. 4:*

**Figure:**